# Comparative Analysis of Novel Lytic Phages for Biological Control of Phytopathogenic *Xanthomonas* spp.

**DOI:** 10.1128/spectrum.02960-22

**Published:** 2022-11-03

**Authors:** M. L. Domingo-Calap, M. Bernabéu-Gimeno, C. M. Aure, E. Marco-Noales, P. Domingo-Calap

**Affiliations:** a Tragsa, Empresa de Transformación Agraria, Valencia, Spain; b Centro de Protección Vegetal y Biotecnología, Instituto Valenciano de Investigaciones Agrarias (IVIA), Moncada, Spain; c Instituto de Biología Integrativa de Sistemas (I^2^SysBio), Universitat de València-CSIC, Paterna, Spain; Institut Pasteur

**Keywords:** plant-pathogenic bacteria, phage, biocontrol, *Xanthomonas* spp.

## Abstract

*Xanthomonas* is an important genus of plant-pathogenic bacteria that affects agronomic and economically important crops, causing serious economic losses. In fact, several *Xanthomonas* species are considered regulated quarantine pests. Due to the lack of effective control measures to treat plant-pathogenic bacteria, innovative control tools are needed to carry out integrated disease management. In this regard, bacteriophages (phages), viruses of bacteria, constitute a promising biocontrol tool. In this work, we report the isolation and characterization of 11 novel Xanthomonas arboricola pv. *juglandis* phages belonging to different families and genera of the class *Caudoviricetes*. Infectivity matrix in more than 60 isolates of different xanthomonads and other phytopathogenic bacteria suggests that these phages are specific to the *Xanthomonas* genus, with different host ranges depending on the isolates tested. Interestingly, some of these phages showed relevant features to be used as biocontrol tools to combat pathogenic *Xanthomonas* spp. as important as *X. oryzae* or *X. citri*.

**IMPORTANCE** Phytopathogenic bacteria represent serious losses worldwide. The lack of current treatments has focused the spotlight on phages, viruses of bacteria, as very promising biocontrol tools. Phages are very specific and can help to control bacterial infections in crops, as is the case of xanthomonads-associated diseases. The discovery of new environmental phages with lytic capacity that can help to combat these pathogens is of special relevance, and it is necessary to implement phage isolation and characterization techniques to determine their host range and their genomic properties. The establishment of phage collections worldwide will allow their use as preventive, diagnostic, or therapeutic tools. Although there is still a long way to go, this work is a step forward in the implementation of new ecofriendly techniques to combat key pathogens in the field.

## INTRODUCTION

Plant diseases are a major cause of production losses worldwide, with important economic, social, and ecological consequences. In particular, plant-pathogenic bacteria are difficult to manage due to their recurrence, rapid bacterial dissemination in crops, and lack of effective control methods (1, 2). The maintenance of crop production systems involves the identification and deployment of sustainable solutions for bacterial disease management, including biocontrol strategies (3). In general, the control of bacterial diseases in crops consists of cultural practices aimed at reducing the bacterial population together with the application of copper compounds and antibiotics where allowed (4, 5). Unfortunately, copper, due to its toxicity, has negative effects on agricultural systems and the environment. In addition, its bioaccumulation in soil and surface water reduces microbial biodiversity (6). The most commonly used antibiotics in agriculture, such as streptomycin, kasugamycin, and tetracycline, may also pose unacceptable risks when used as pesticides (3), and their use is not allowed in many countries. Moreover, copper compounds and antibiotics lead to the emergence of resistance in bacteria. All this has raised worldwide interest in research and application of biocontrol for the management of bacterial plant diseases.

In this context, the use of bacteriophages, bacterial viruses, is one of the most promising ecofriendly alternatives to combat phytopathogenic bacteria. Phages have important advantages over chemical compounds. Their natural presence in all ecosystems does not pose a danger to the environment since their use is considered a minor intervention in the native microbiota of the plant (6). In fact, the application of phages could contribute to maintaining beneficial bacteria by suppressing target pathogenic bacteria. Other benefits are their low cost of production, their autoamplification (autodosage), and their ability to counteract bacterial resistance (7).

Among phytopathogenic bacteria, the genus *Xanthomonas* stands out for containing a large number of species (more than 35) pathogenic to more than 400 plant species (8). In fact, xanthomonads are the largest group of plant bacteria that cause devastating diseases in crops and are considered a global threat (6). The species Xanthomonas arboricola has been proposed to be composed of nine pathovars, and some of them are the most important phytopathogenic bacteria of stone fruits and nuts (9). Among them, X. arboricola pv. *juglandis* is the causal agent of walnut blight disease, a severe disease of walnuts that can produce serious damage in leaves, nuts, buds, catkins, and young twigs and is considered the most serious biotic stress affecting Persian walnut trees, causing economic losses in walnut production worldwide (10). Information on the detection of X. arboricola pv. *juglandis* strains has been increasing throughout Europe, and population studies have found an unprecedented genetic diversity of this pathovar (8). Infected nursery stock is considered the main route of introduction and spread in newly cultivated areas, although it has been shown that it can be disseminated through pollen (9). Moreover, globalization and plant trade favor its dispersal worldwide, and global warming allows the pathogen to adapt and establish in new areas (6).

The first use of phages as biocontrol agents against *Xanthomonas* spp. dates from the 19th century (11). Since then, more than 160 phages specific for different xanthomonads have been isolated worldwide (12), belonging to 5 families: *Podoviridae*, *Siphoviridae*, *Myoviridae*, *Autographiviridae*, and *Herelleviridae* (12). These last two families collect the lowest number of phages infecting *Xanthomonas* spp., and the family *Autographiviridae* includes only 3 members, none of them infecting X. arboricola pv. *juglandis*. In this regard, all the reported phages infecting the strains of this pathovar belong to the families *Podoviridae* and *Siphoviridae* (13, 14). Recently, novel phages against important pathogenic species have been reported, including Xanthomonas citri (15, 16), Xanthomonas translucens, and Xanthomonas campestris (17) and Xanthomonas oryzae (18). To date, two *Xanthomonas* phage-based products are commercially available for biological control (manufactured by AgriPhage [https://www.omnilytics.com/]) and have been shown to successfully control pathogens that cause tomato and pepper spot disease and citrus canker disease. Despite this, there is great unexploited potential for the use of phages to replace or reduce the number of agrochemicals used for control of most plant-pathogenic bacteria.

In the present work, we have isolated and characterized 11 novel bacteriophages against the pathogen X. arboricola pv*. juglandis* from environmental samples in Valencia (Spain). All phages were morphological and genomically characterized and showed high diversity. Host range analysis on a large collection of pathogenic *Xanthomonas* spp. and other phytopathogenic bacteria showed that the isolated phages were highly specific against *Xanthomonas* spp. and had strong lytic activity against some quarantine bacterial strains of species that constitute major threats for agriculture. This work provides an excellent starting point for investigating the potential use of these phages in biocontrol programs to combat xanthomonads worldwide.

## RESULTS

### Phage isolation and purification.

A total of 11 X. arboricola pv. *juglandis* phages against the host IVIA 1317-1a, named vB_Xar_IVIA-DoCa1 to vB_Xar_IVIA-DoCa11, were isolated from sewage using the double-layer agar technique. To isolate each phage, a triple plaque-to-plaque transfer was performed. Subsequently, each isolated plaque was used to infect log-phase bacterial cultures, and the supernatant was titered by the standard plaque assay. Under these conditions, the viral titer of the isolated phages lysates ranged from 10^9^ to 10^10^ PFU/mL on lawns of IVIA 1317-1a. All phages formed clear plaques, with sizes ranging from 0.5 to 4.0 mm in diameter and well-defined boundaries.

### Transmission electron phage micrographs.

**FIG 1:**
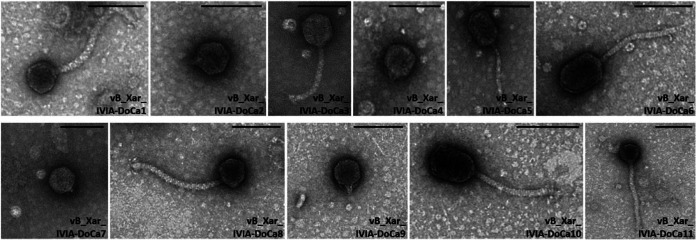
Morphological characterization of the isolated *Xanthomonas* phages. Transmission electron microscopy images are shown; scale bars, 100 nm.

Transmission electron micrographs from high-titer phage-lysed cultures revealed three main phage morphologies (Fig. 1). Phages vB_Xar_IVIA-DoCa2, vB_Xar_IVIA-DoCa4, vB_Xar_IVIA-DoCa7, and vB_Xar_IVIA-DoCa9 showed an isometric head (diameter of approximately 50 nm) and a very short cone-shaped tail (length of approximately 10 nm). Phages vB_Xar_IVIA-DoCa1, vB_Xar_IVIA-DoCa3, vB_Xar_IVIA-DoCa8, and vB_Xar_IVIA-DoCa11 had larger sizes with an isometric head (diameter of approximately 50 nm) and a long cone-shaped tail (length greater than 100 nm). Finally, phages vB_Xar_IVIA-DoCa5, vB_Xar_IVIA-DoCa6, and vB_Xar_IVIA-DoCa10 had icosahedral elongated heads (diameter of approximately 70 nm) and a long cone-shaped tail (length greater than 100 nm).

### Lytic activity of the isolated phages in IVIA 1317-1a cultures.

**FIG 2:**
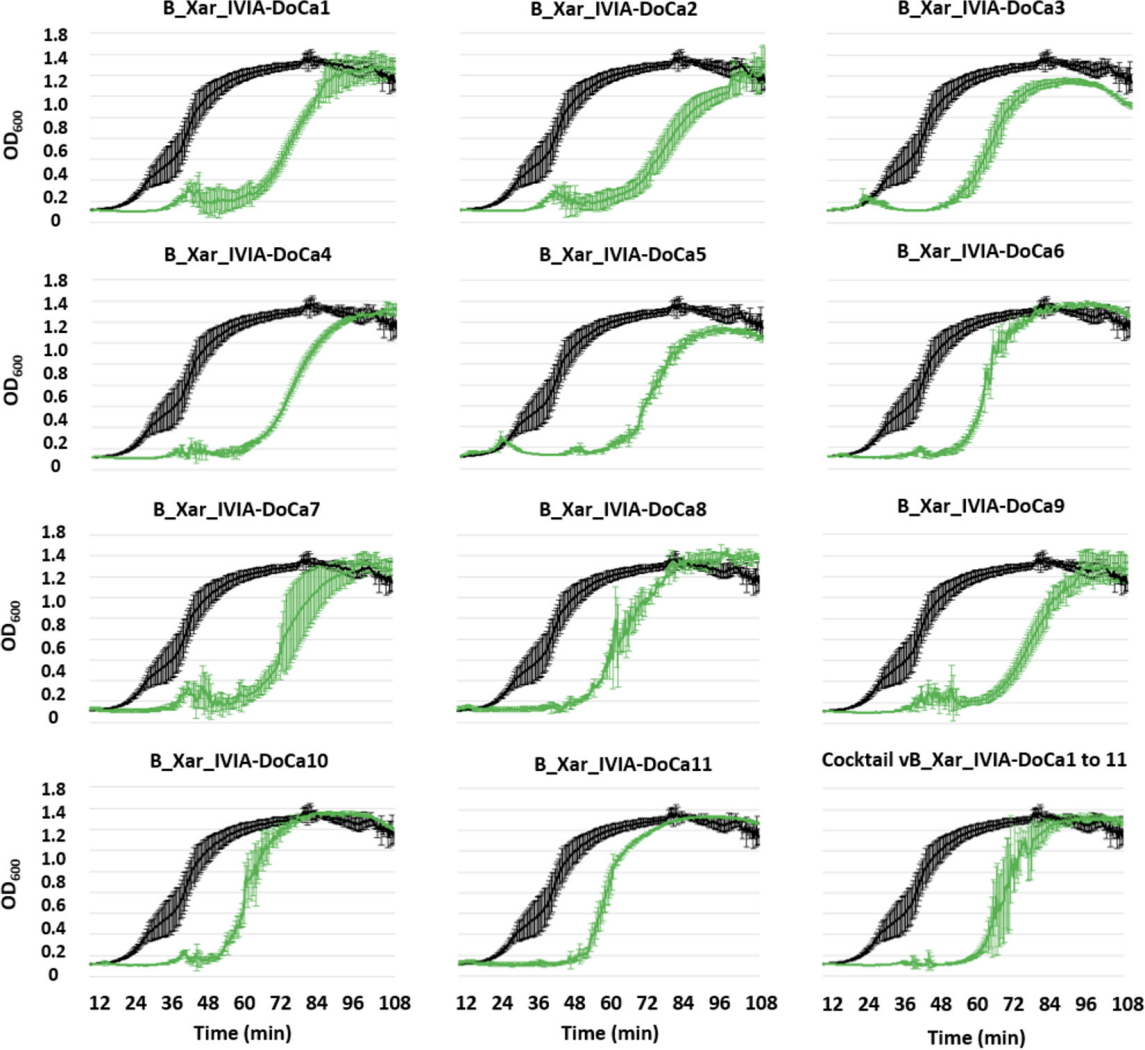
Growth curves of strain IVIA 1317-1a in the presence/absence of the isolated *Xanthomonas* phages. In total, 11 phages named vB_Xar_IVIA-DoCa1 to vB_Xar_IVIA-DoCa11 are shown. In addition, a cocktail encompassing the 11 phages was tested. Each measured point was recorded every 30 min until 110 h. All the experiments were done in triplicate, and error bars are shown. Black indicates the absence of phage, and green indicates the presence of phage.

To assess the lytic activity of the isolated phages in liquid medium, log-phase IVIA 1317-1a cultures were inoculated with each phage in parallel with noninfected cultures grown as controls (Fig. 2). In the presence of each phage, a drastic reduction in growth of bacterial strain IVIA 1317-1a was observed during the first 48 h, followed by the emergence of bacterial resistance clones in all cases. However, the timing of the emergence of resistance varied slightly, depending on the phage, between 42 h and around 60 h postinfection. In addition, a phage cocktail encompassing all the isolated phages (vB_Xar_IVIA-DoCa1 to vB_Xar_IVIA-DoCa11) did not prevent the emergence of resistance, although resistance emergence was delayed until after 60 h. These assays revealed a strong lytic effect, as the growth of the bacterial strain was completely inhibited until the emergence of resistant clones, as also suggested by the large and clear plaques formed in bacterial lawns.

### Determination of phage host range.

**FIG 3:**
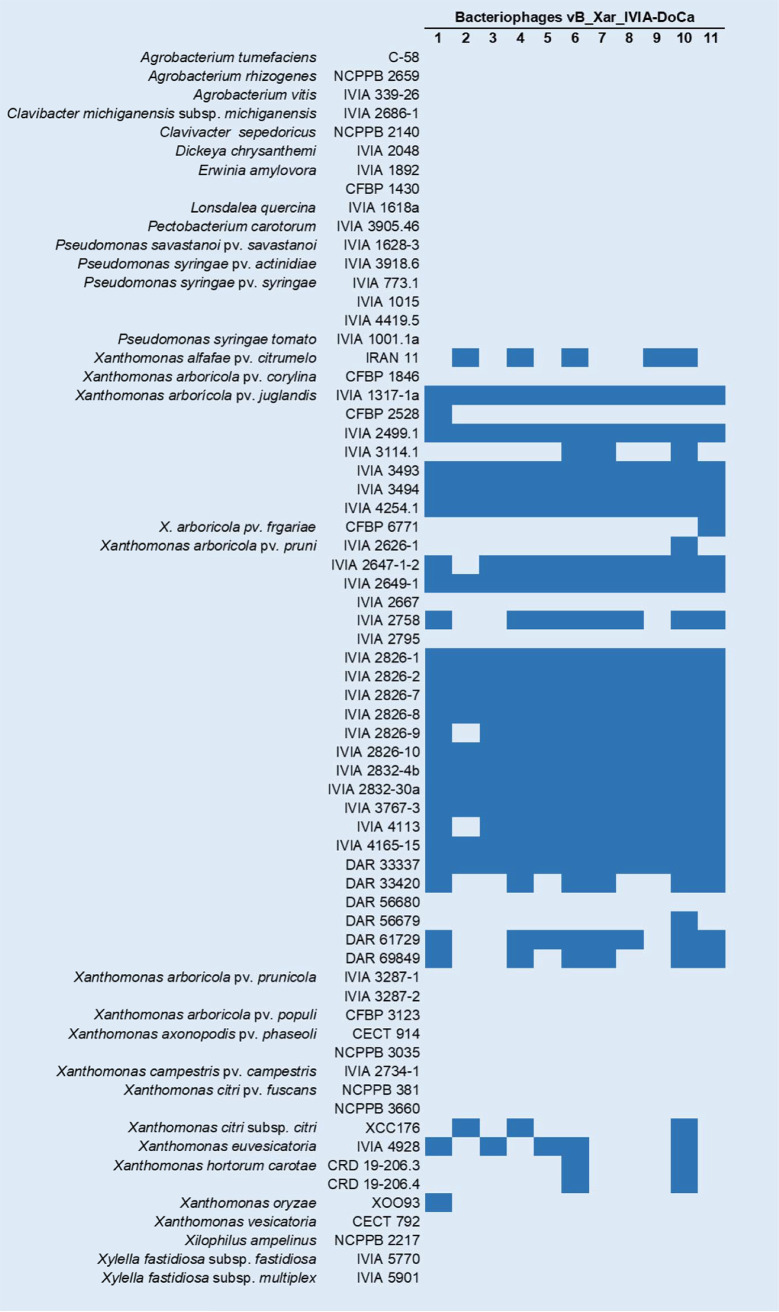
Host range of the isolated *Xanthomonas* phages against 47 strains of *Xanthomonas* spp. and 19 strains of other phytopathogenic bacteria. In total, 11 phages named vB_Xar_IVIA-DoCa1 to vB_Xar_IVIA-DoCa11 are shown; blue, lysis; white, no lysis.

To determine the host range of the new X. arboricola pv. *juglandis* phages, spot tests in soft agar plates were performed for a battery of 63 bacterial isolates, including 46 *Xanthomonas* species isolates and 19 distant phytopathogenic bacterial species (Fig. 3). The phages showed lytic activity against some *Xanthomonas* species strains, including quarantine species such as X. oryzae and X. citri, with different ranges depending on the phage considered. In the case of isolates of other phytopathogenic bacterial genera, none of them were susceptible to viral infection by any of the new isolated phages.

Focusing on X. arboricola pv. *juglandis*, six independent isolates were used to determine the host range of the phages, and four isolates were susceptible to all phages. In contrast, isolate CFBP 2528 was susceptible only to vB_Xar_IVIA-DoCa1, and isolate IVIA 3114.1 was susceptible only to vB_Xar_IVIA-DoCa6, vB_Xar_IVIA-DoCa7, and vB_Xar_IVIA-DoCa10. Regarding the whole infectivity matrix, which included several *Xanthomonas* pathovars and species, phage vB_Xar_IVIA-DoCa10 was the phage showing the broadest host range, infecting 31 of the evaluated strains. In contrast, vB_Xar_IVIA-DoCa2 was the phage with the narrowest host range and was able to infect only 18 of the isolates. Indeed, some *Xanthomonas* species isolates were not infected by any of the 11 phages: CFBP 1846 of X. arboricola pv. *corylina*, strains IVIA 2667, IVIA 2795, and DAR 56680 of X. arboricola pv. *pruni*, strains IVIA 3287-1 and IVIA 3287-2 of X. arboricola pv. *prunicola*, strain CFBP 3123 of X. arboricola pv. *populi*, strains CECT914 and NCPPB 3035 of *X. axonopodis* pv. *phaseoli*, strain IVIA 2734-1 of X. campestris pv. *campestris*, strains NCPPB 381 and NCPPB 3660 of *X. citri* pv. *fuscans*, and strain CECT792 of *X. vesicatoria*.

### Genome sequencing and comparative genomics of the phages.

The 11 phages were sequenced using the MiSeq Illumina platform. The sequencing run led to 685,328 to 5,808,456 filtered paired-end reads (depending on the phage) that were used to perform the *de novo* assembly with SPAdes v3.13.1 (19). One long contig with high coverage was found in all the samples, indicating the assembly of complete phage genomes. These genomes were then reorganized based on the start of the large terminase subunit and corrected with Pilon. The resulting genomes had sizes ranging from 40 to 60 kb, with a G+C content fluctuating between 54 and 66% (Table 1).

**TABLE 1:** General features of phage genomes of vB_Xar_IVIA-DoCa1 to vB_Xar_IVIA-DoCa11, including size, percentage of GC and coverage of the assembled genomes, predicted ORFs by consensus gene calling, and most related phage in the nucleotide database by BLASTn analysis

*Xanthomonas* phage	Size (bp)	GC content (%)	Avg sequencing coverage	No. of CDS^*a*^	BLAST nearest genome				Class	Family^*b*^	Genus^*b*^
					Name	Cov.^*a*^ (%)	E value	Ident.^*a*^ (%)			
vB_Xar_IVIA-DoCa1	43,533	54.4	211	54	*Xanthomonas* phage Samson	94	0.0	98.47	*Caudoviricetes*	*-*	*Septimatrevirus*
vB_Xar_IVIA-DoCa2	42,846	62.7	1,914	51	*Xylella* phage Prado	88	0.0	96.06	*Caudoviricetes*	*Autographiviridae*	*Pradovirus*
vB_Xar_IVIA-DoCa3	58,103	58.6	3,532	75	Pseudomonas phage JG012	94	0.0	96.89	*Caudoviricetes*	*-*	*Nipunavirus*
vB_Xar_IVIA-DoCa4	42,996	62.9	593	50	*Xylella* phage Prado	94	0.0	95.89	*Caudoviricetes*	*Autographiviridae*	*Pradovirus*
vB_Xar_IVIA-DoCa5	56,407	59.6	1,209	74	*Stenotrophomonas* phage vB_Sm_QDWS359	42	0.0	77.59	*Caudoviricetes*	*Mesyanzhinoviridae*	*Bosavirus*
vB_Xar_IVIA-DoCa6	62,950	67.0	275	82	*Xanthomonas* phage Bosa	97	0.0	93.51	*Caudoviricetes*	*Mesyanzhinoviridae*	*Bosavirus*
vB_Xar_IVIA-DoCa7	42,843	59.4	2,451	55	*Stenotrophomonas* phage Ponderosa	4	0.0	81.97	*Caudoviricetes*	*Autographiviridae*	*-*
vB_Xar_IVIA-DoCa8	43,677	54.4	6,247	55	Pseudomonas phage vB_Pae_TR	91	0.0	98.48	*Caudoviricetes*	*-*	*Septimatrevirus*
vB_Xar_IVIA-DoCa9	43,229	62.9	3,792	49	*Xylella* phage Prado	91	0.0	96.09	*Caudoviricetes*	*Autographiviridae*	*Pradovirus*
vB_Xar_IVIA-DoCa10	64,023	65.5	379	84	*Xanthomonas* phage Bosa	97	0.0	94.43	*Caudoviricetes*	*Mesyanzhinoviridae*	*Bosavirus*
vB_Xar_IVIA-DoCa11	43,005	53.7	1,387	56	Pseudomonas phage vB_Pae_PS9N	97	0.0	98.01	*Caudoviricetes*	*-*	*Septimatrevirus*

a CDS, coding sequence; cov., coverage; indent., identity.

b -, taxonomic rank not determined or under discussion by the International Committee on Taxonomy of Viruses (ICTV).

Comparison against the nucleotide database using BLAST (20) showed a closest relative with high coverage and similarity for all the phages except for vB_Xar_IVIA-DoCa5 and vB_Xar_IVIA-DoCa7. In these two phages, only 42% and 4% of their genomes, respectively, were similar to a genome available in public databases. In vB_Xar_IVIA-DoCa7, 4% corresponded to regions encoding a 3′ to 5′ exonuclease, a major capsid protein, and a DNA polymerase. The nearest organisms to all phages belonged to different families and genera of the class *Caudoviricetes*; vB_Xar_IVIA-DoCa2, vB_Xar_IVIA-DoCa4, vB_Xar_IVIA-DoCa7, and vB_Xar_IVIA-DoCa9 showed similarities with phages from the *Autographiviridae* family, while vB_Xar_IVIA-DoCa1, vB_Xar_IVIA-DoCa3, vB_Xar_IVIA-DoCa5, vB_Xar_IVIA-DoCa6, vB_Xar_IVIA-DoCa8, vB_Xar_IVIA-DoCa10, and vB_Xar_IVIA-DoCa11 were more similar to phages from the *Septimatrevirus*, *Nipunavirus*, and *Bosavirus* genera. Specifically, the closest relatives were of the genus *Pradovirus* for vB_Xar_IVIA-DoCa2, vB_Xar_IVIA-DoCa4, and vB_Xar_IVIA-DoCa9, *Septimatrevirus* for vB_Xar_IVIA-DoCa1, vB_Xar_IVIA-DoCa8, and vB_Xar_IVIA-DoCa11, *Nipunavirus* for vB_Xar_IVIA-DoCa3, and *Bosavirus* for vB_Xar_IVIA-DoCa5, vB_Xar_IVIA-DoCa6, and vB_Xar_IVIA-DoCa10.

### Functional annotation, comparative analysis, and lifestyle prediction of phages.

Consensus gene calling using Phanotate (21), Prodigal (22), and Glimmer (23) identified around 50 open reading frames (ORFs) in phages belonging to the *Autographiviridae* family and *Septimatrevirus* genus, while more than 70 ORFs were found in all phages belonging to the *Nipunavirus* and *Bosavirus* genera. BLAST (20) against the RefSeq protein database allowed the functional annotation of almost all the ORFs identified except for those from the vB_Xar_IVIA-DoCa7 genome that maintain 48 of 55 predicted ORFs without functional annotation. CD-Search (24) using the Conserved Domains Database (CDD) detected conserved protein domains in 16 of these 48 ORFs without functional annotation predicted in the vB_Xar_IVIA-DoCa7 genome. Moreover, 1 of the 9 ORFs without functional annotation predicted in the vB_Xar_IVIA-DoCa2 genome and 1 of the 5 ORFs without functional annotation predicted in the vB_Xar_IVIA-DoCa6 genome had domain hits. The rest of the ORFs identified remain without functional annotation.

**FIG 4:**
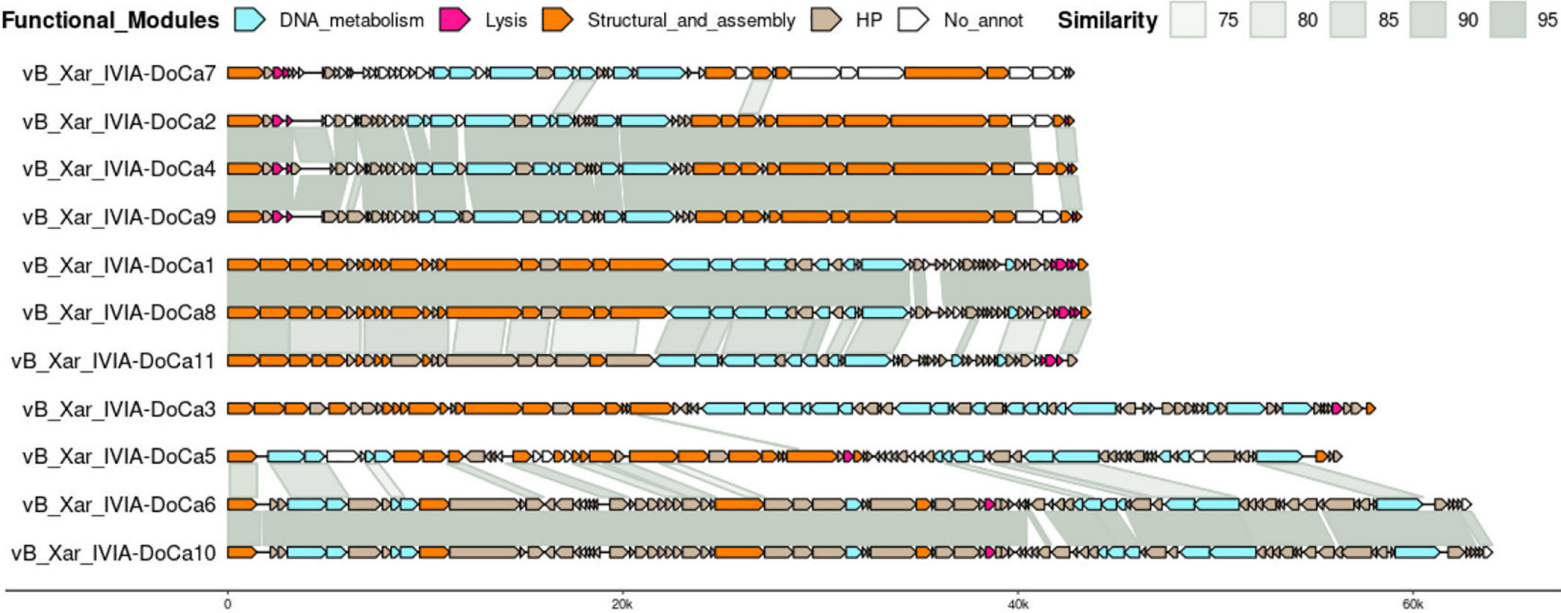
Comparative genomic organization of the isolated *Xanthomonas* phages. In total, 11 phages named vB_Xar_IVIA-DoCa1 to vB_Xar_IVIA-DoCa11 are shown. Predicted ORFs by the consensus of gene callers Glimmer3, Phanotate, and Prodigal are represented by arrows colored based on their function, predicted using BLASTp against the RefSeq viral protein database and CD-Search using CDD. No annotated ORFs were left in white. Representations were generated using gggenomes (48). Overlapping between the first and last gene in all the genomes is not shown.

All phages showed a genomic organization previously described in phages from the same genus (Fig. 4). Similar to *Xylella* phage Prado (accession number NC_022987), vB_Xar_IVIA-DoCa2, vB_Xar_IVIA-DoCa4, and vB_Xar_IVIA-DoCa9 are phiKMV-like phages with genomes consisting of three functional modules, with a gene encoding a single-subunit RNA polymerase at the end of the second cluster formed by genes involved in DNA metabolism (25). In the case of VB_Xar_IVIA-DoCa7, despite having half of the predicted ORFs without functional annotation, similar genomic organization could be identified. Genes of vB_Xar_IVIA-DoCa1, vB_Xar_IVIA-DoCa8, and vB_Xar_IVIA-DoCa11 were also grouped in functional clusters. All of these genes encoded a RecB exonuclease and a RecA ATPase, homologous recombination proteins present in genomes from other phages belonging to the *Septimatrevirus* genus, like the Pseudomonas phage vB_Pae-Kakheti25 (accession number JQ307387) (26). vB_Xar_IVIA-DoCa1 and vB_Xar_IVIA-DoCa8 showed a similarity higher than 95% in almost their entire genome, whereas vB_Xar_IVIA-DoCa11 was slightly different. vB_Xar_IVIA-DoCa3 has a genomic organization similar to Pseudomonas phages vB_PaeS_PAJD-1 (accession number MW835180) and Pamx25 (accession number NC_041953), NP1-like phages belonging to the *Nipunavirus* genus (27, 28). In addition to an ORF encoding a RecB exonuclease, a cluster of ORFs related to the biosynthesis of 7-deazaguanine derivatives (ORFs 30 to 34) and an ORF encoding a UvrD helicase were found, which have been described as essential for the replication of filamentous phages (29). vB_Xar_IVIA-DoCa6 and vB_Xar_IVIA-DoCa10 had the largest genomes with more than 80 ORFs. Both showed high similarity to each other and to the temperate phages belonging to the *Bosavirus* genus, *Xanthomonas* phage Bosa (accession number NC_052967), and Stenotrophomonas maltophilia phage DLP4 (accession number MG018224). VB_Xar_IVIA-DoCa5 was more different than VB_Xar_IVIA-DoCa6 and VB_Xar_IVIA-DoCa10 (and to phages previously described) but had a modular arrangement of genes similar to those and *Xanthomonas* phage Bosa (30) and S. maltophilia phage DLP4 (31). Interestingly, in vB_Xar_IVIA-DoCa5, vB_Xar_IVIA-DoCa6, and vB_Xar_IVIA-DoCa10, a gene very similar to a putative integrase gene predicted in the genome of *Xanthomonas* phage Bosa, and renamed as “bifunctional primase/polymerase” in the RefSeq database (ORF 71, ORF 77, and ORF 78, respectively), was detected.

To determine the relationship between the isolated phages and those previously described, unclassified virus belonging to the family *Autographiviridae* and phages belonging to the subfamily *Bradleyvirinae* and the genera *Pradovirus*, *Nipunavirus*, and *Septimatrevirus* were selected and used to perform a single-gene phylogenetic analysis and a whole-genome comparison.

**FIG 5:**
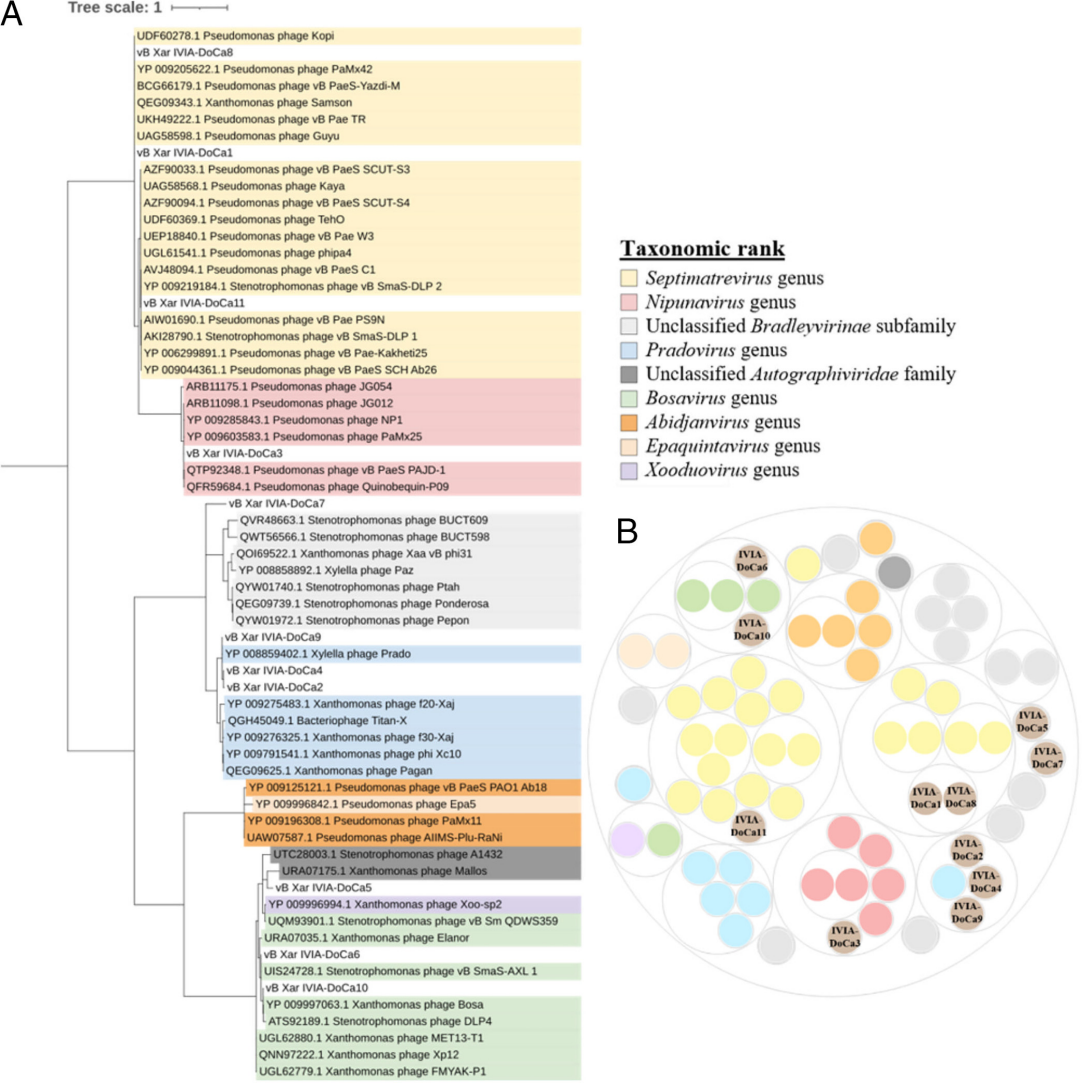
Taxonomy of the isolated *Xanthomonas* spp. phages. (A) Maximum likelihood phylogenetic tree based on DNA polymerase protein sequences of the phages. Root is fixed at midpoint, and all branches have a bootstrap value of at least 90. (B) VIRIDIC clustering by intergenomic similarity (threshold of 95% and 70% for the demarcation of viruses into species and genera). In total, 11 phages named vB_Xar_IVIA-DoCa1 to vB_Xar_IVIA-DoCa11 are shown, including previously published phage species of unclassified virus belonging to the family *Autographiviridae* and phages belonging to the subfamily *Bradleyvirinae* and the genera *Pradovirus*, *Nipunavirus*, and *Septimatrevirus*.

First, a maximum likelihood phylogeny was performed with the sequences of the DNA polymerase protein using the VT substitution model, grouping the phages into different taxonomic ranks (Fig. 5A). On one hand, VB_Xar_IVIA-DoCa1, VB_Xar_IVIA-DoCa8, and VB_Xar_IVIA-DoCa11 were close to phages belonging to the *Septimatrevirus* genus, and VB_Xar_IVIA-DoCa3 was closer to phages belonging to the *Nipunavirus* genus. VB_Xar_IVIA-DoCa5, VB_Xar_IVIA-DoCa6, and VB_Xar_IVIA-DoCa10 fell within phages belonging to the *Bradleyvirinae* subfamily, even though VB_Xar_IVIA-DoCa6 and VB_Xar_IVIA-DoCa10 were more closely related, and to other phages of the *Bosavirus* genus. On the other hand, VB_Xar_IVIA-DoCa4, VB_Xar_IVIA-DoCa2, VB_Xar_IVIA-DoCa7, and VB_Xar_IVIA-DoCa9 were grouped with phages of the *Autographiviridae* family, although VB_Xar_IVIA-DoCa2, VB_Xar_IVIA-DoCa4, and VB_Xar_IVIA-DoCa9 were closer to each other, and to other phages belonging to the *Pradovirus* genus. As previously shown by other methods, VB_Xar_IVIA-DoCa7 appeared to be the most distant phage.

Second, fixing a threshold of 95% for the demarcation of viruses into species and 70% into genera, as suggested by the International Committee on Taxonomy of Viruses (ICTV), whole-genome sequencing comparison was done by VIRIDIC. Nine of the 11 phages isolated clustered with previously described phages (at least at the genus level), similar to the results from single-gene phylogeny. However, vB_Xar_IVIADoCa5 and vB_Xar_IVIA-DoCa7 remained unclustered (Fig. 5B). These results are understandable since only 42% and 7% of their genomes, respectively, were similar to genomes available in public databases. Interestingly, these two phages seem to be related to other phages isolated in this study, vB_Xar_IVIADoCa6 and vB_Xar_IVIADoCa10 in the case of vB_Xar_IVIADoCa5 (50% of intergenomic similarity) and vB_Xar_IVIADoCa2, vB_Xar_IVIADoCa4, and vB_Xar_IVIADoCa9 in the case of vB_Xar_IVIA-DoCa7 (23% of intergenomic similarity). Moreover, this analysis showed that phages vB_Xar_IVIA-DoCa2, vB_Xar_IVIA-DoCa4, and vB_Xar_IVIA-DoCa9 belong to the same genera but not to the same species (similarity from 89.7 to 92.2% in all the pairwise comparisons). In contrast, vB_Xar_IVIA-DoCa1 and vB_Xar_IVIA-DoCa8 belong to the same species (intergenomic similarity of 96%) and these along with vB_Xar_IVIA-DoCa11 belong to the same genera (intergenomic similarity of 70% in all the pairwise comparisons). Finally, vB_Xar_IVIA-DoCa6 and vB_Xar_IVIA-DoCa10 belong to the same genera (intergenomic similarity of 90.6%). A complete heat map was obtained with VIRIDIC (Fig. S1 in the supplemental material).

Despite the similarity of vB_Xar_IVIA-DoCa5, vB_Xar_IVIA-DoCa6, and vB_Xar_IVIA-DoCa10 with temperate phages belonging to the *Bosavirus* genus and the detection of a gene very similar to a putative integrase gene predicted in the genome of *Xanthomonas* phage Bosa, lifestyle prediction by PhageAI (32) and Bacphlip (33) showed them to be virulent (Table 2). Specifically, vB_Xar_IVIA-DoCa10 was predicted to be virulent with a probability higher than 85% by both software packages, whereas PhageAI and Bacphlip differ slightly more when analyzing vB_Xar_IVIA-DoCa5 and vB_Xar_IVIA-DoCa6.

**TABLE 2:** Lifestyle prediction of phages vB_Xar_IVIA-DoCa1 to vB_Xar_IVIA-DoCa11 and probability of vB_Xar_IVIA-DoCa1 to vB_Xar_IVIA-DoCa11 phages being virulent as assessed by two different software packages of lifestyle prediction (PhageAI and Bacphlip)

*Xanthomonas* phage	PhageAI prediction	Bacphlip prediction
vB_Xar_IVIA-DoCa1	Virulent (100.00%)	Virulent (96.15%)
vB_Xar_IVIA-DoCa2	Virulent (88.00%)	Virulent (52.50%)
vB_Xar_IVIA-DoCa3	Virulent (93.47%)	Virulent (100.00%)
vB_Xar_IVIA-DoCa4	Virulent (88.30%)	Virulent (98.75%)
vB_Xar_IVIA-DoCa5	Virulent (66.64%)	Virulent (95.00%)
vB_Xar_IVIA-DoCa6	Virulent (99.21%)	Virulent (78.75%)
vB_Xar_IVIA-DoCa7	Virulent (93.47%)	Virulent (98.75%)
vB_Xar_IVIA-DoCa8	Virulent (100%)	Virulent (99.17%)
vB_Xar_IVIA-DoCa9	Virulent (89.46%)	Virulent (89.50%)
vB_Xar_IVIA-DoCa10	Virulent (99.33%)	Virulent (86.25%)
vB_Xar_IVIA-DoCa11	Virulent (100.00%)	Virulent (85.00%)

## DISCUSSION

In the last years, reports on phage biocontrol of plant-pathogenic bacteria are arising (6, 34, 35). However, there is still a long way to reduce the use of agrochemicals and to integrate phage as a valuable tool in management programs. The great biodiversity of phages in nature, being the most variable and abundant biological entities in the biosphere, makes very different niches an important source of phages. The isolation of new phages from the environment will help to implement novel strategies to combat pathogenic bacteria, as an ecofriendly alternative, preserving the microbiome of the plant ecosystem and leaving no residues. This approach requires specific protocols for phage hunting and characterization of novel viruses and an understanding of the emergence of phage-resistant clones and the infectivity of other bacterial species. In the present work, we have isolated and characterized 11 X. arboricola pv*. juglandis* phages, vB_Xar_IVIA-DoCa1 to vB_Xar_IVIA-DoCa11, with strong lytic activity against several plant-pathogenic *Xanthomonas* species, causal agents of many important crop diseases that produce economically significant losses worldwide. All the phages were isolated from wastewater samples from the metropolitan area of Valencia (Spain), and an isolate X. arboricola pv*. juglandis* was used as the primary host. After purification of the phages that showed different plaque morphology or from different sampling times, in depth characterization of the phages was performed, including phenotypic and genomic characterization. vB_Xar_IVIA-DoCa7 was the most divergent phage, showing only 4% similarity with previously reported viruses. The closest phage, within the *Pradovirus* genus, included interesting phages capable of lysing the major pathogen Xylella fastidiosa (25). However, our results suggested that any of the isolated *Xanthomonas* phages were able to lyse X. fastidiosa strains.

Previous work isolating phages against X. arboricola pv. *juglandis* reported 26 phages from plant material, all belonging to the *Podoviridae* and *Siphoviridae* families by electron micrographs but with no genomic data available (14). All of them were capable of infecting bacterial species other than *Xanthomonas* spp., suggesting broad-range infection and reducing their potential use as biocontrol agents. Recently, 24 phages were isolated from soil samples and infected walnut plants, but only two of them were selected for characterization, and no infectivity test in other bacteria was done (13). Again, one of these phages belonged to the *Podoviridae* family and the other belonged to the *Siphoviridae* family. Here, we have isolated a total of 11 phages from wastewater samples, and 4 of them (vB_Xar_IVIA-DoCa2, vB_Xar_IVIA-DoCa4, vB_Xar_IVIA-DoCa7, and vB_Xar_IVIA-DoCa9) belong to the *Autographiviridae* family. So far, these phages represent the first phages isolated against X. arboricola pv. *juglandis* belonging to a different family. Moreover, all of these phages are highly specific, since they have not been able to infect any of the 19 isolates of different phytopathogenic bacteria tested, which makes them good candidates for use in biocontrol of walnut blight disease.

Phage biocontrol requires the use of lytic phages to combat pathogenic bacteria without disturbing the microbiome of the plant and reducing secondary effects in the plant and the ecosystem (36). Lysogenic phages can transduce genetic material from one bacterium to another and have implications in terms of resistance or other virulence factors (37). For these reasons, it is important to detect potential proteins involved in lysogeny in phage genomes. In this regard, vB_Xar_IVIA-DoCa5, vB_Xar_IVIA-DoCa6, and vB_Xar_IVIA-DoCa10 encode a putative integrase, suggesting that they may be capable of lysogenic conversion. However, lifestyle prediction of these phages with two different software packages suggested that they are strictly lytic, as observed in semisolid and liquid cultures, in terms of plaque formation and inhibition of growth. In addition, the characterization of the cyanophage S-TIP37 demonstrated that the presence of an integrase does not imply a lysogenic life cycle (38). Despite these results, further research in this direction should be done to determine the lysogenic potential of the phages and the actual involvement of temperate phase in the life cycle of these phages.

Another important feature of phages for biocontrol is their specificity, usually limited to certain strains within a particular bacterial species. However, some polyvalent phages are capable of infecting different species within the same genus (39), as is the case of the phages isolated in this study, with vB_Xar_IVIA-DoCa10 being the phage with the broadest range. Nevertheless, differences in the host range of the phages within the xanthomonads group suggest variability within phages, as previously reported (13, 14). This diversity in phage response is not surprising considering the enormous genetic diversity of *Xanthomonas* spp. In fact, high genome plasticity has been demonstrated in several xanthomonads, pointing to mechanisms of adaptation and selection (8). Differences in cell wall receptors in susceptible and phage-resistant strains for the phages may be responsible, although other factors, such as postentry resistance mechanisms, may also be involved, which requires further investigation. Although phage biocontrol can be used as an *ad hoc* strategy to specifically control a given pathogenic bacterium, the phages isolated in the present study are able to lyse bacteria belonging to the same pathovar from different origins, suggesting the potential of these phages to control multiple *Xanthomonas* strains over the world. Interestingly, none of the nonxanthomonad bacteria tested were susceptible to the phages, indicating a genus specificity that could be useful for biocontrol of xanthomonads.

To develop a phage biocontrol tool, phage cocktails are an interesting solution to broaden the spectrum of action and also counteract bacterial resistance. Cocktails should contain phages capable of recognizing different strains, with the objective of constituting a useful strategy to control pathogenic bacteria (40). Thus, a phage cocktail could be designed based on the range host matrix obtained in this work to specifically control different pathogenic *Xanthomonas* spp. The other possible function of phage cocktails is to reduce or delay the emergence of bacterial phage-resistant clones, although it has been shown that some resistant clones are avirulent *in vivo* (41, 42). Here, we have tested a phage cocktail encompassing the 11 phages in the primary bacterial host in the bacterium used for phage hunting to prevent the emergence of resistant clones. In fact, the cocktail delayed the emergence of phage-resistant clones beyond 60 h. However, future *in vivo* analysis might determine differences in resistance emergence or in infectivity using other phytopathogenic hosts.

### Conclusions.

Although much research remains to be done to develop a phage biocontrol product against phytopathogenic xanthomonads, this work represents a step forward in the understanding of phage diversity and infectivity. Here, 11 *Xanthomonas* phages with strong lytic activity against plant-pathogenic species have been characterized in depth. Their potential as biocontrol agents is still incipient, but their properties in terms of host range and lytic activity place them as a promising tool. In addition, phages can be used as diagnostic tools, and bacterial typing based on phage specificity may be another strategy to exploit phages to monitor plant pathogens and adopt appropriate control measures.

## MATERIALS AND METHODS

### Bacterial isolates and growth conditions.

X. arboricola pv. *juglandis* IVIA 1317-1a, isolated from a diseased walnut at Badajoz province (Spain) in 1993, was used as a primary host to isolate phages. Additionally, 46 bacterial isolates belonging to several species of *Xanthomonas* and 19 isolates of other phytopathogenic bacterial species were used (Table S1 in the supplemental material). All bacteria were routinely grown at 25 to 28°C (depending on the bacterial species) in Luria-Bertani (LB) broth under shaking conditions.

### Bacteriophage isolation, purification, amplification, and concentration.

Sewage samples from two wastewater treatment plants from the Valencia metropolitan area (Spain), belonging to the Entidad Pública de Saneamiento de Aguas Residuales (EPSAR) from the Valencian region, were used for phage hunting using strain IVIA 1317-1a as a primary host. Wastewater samples were centrifuged at 3,000 × *g* for 10 min at room temperature and filtered twice through 0.45- and 0.22-μm syringe filters to remove large particles, including bacterial cells and cellular debris. Then, 1-mL aliquots of the filtered samples were added to 100 μL of overnight cultures of the strain IVIA 1317-1a in stationary phase, and the mixture was poured onto petri dishes using the soft agar overlay method. Plates were incubated at 25°C for 24 h to allow phage plaques to develop. Isolated plaques were picked by micropipette aspiration using filter tips, and a triple plaque-to-plaque transfer was performed to generate pure phage isolates. Cleaned phage stocks were kept at 4°C.

Amplification of the phages was performed in liquid cultures of the bacteria. Briefly, 500 μL of IVIA 1317-1a culture at an optical density at 600 nm (OD_600_) of 0.2 were mixed with 10 μL of the cleaned phage stock to amplify it, and the mix was incubated for up to 24 h at 25°C and 250 rpm. Then, cultures with phages were centrifuged (13,000 × *g* for 3 min), and the supernatants were filtered (0.22-μm filter). Lysates were serially diluted and titrated to verify the amplification. To obtain high-titer lysates, amplified phages were filtered through 0.22-μm filters and concentrated by high-speed centrifugation (70,000 × *g* for 2.5 h). For each phage, the pellet was resuspended in 200 μL of SM buffer (50 mM Tris-HCl [pH 7.5], 8 mM MgSO_4_•7H_2_O, 100 mM NaCl, and 0.01% gelatin [w/v]) and kept at −70°C for further analysis.

### Transmission electron microscopy.

To determine phage morphology, purified high-titer phages in SM buffer were analyzed. Briefly, the glow discharge technique (30 s at 7.2 V using a Bal-Tec MED 020 coating system) was applied over carbon-coated copper grids, and grids were immediately placed on top of sample drops for 10 min. After two brief washes in distilled water, samples were contrasted with 2% uranyl acetate for 5 min. Excess fluid was removed and allowed to dry before examination with a transmission electron FEI Tecnai G2 Spirit microscope (Thermo Fisher Scientific, OR, USA). All images were acquired using Radius software (version 2.1) with a digital camera Xarosa (EMSIS, Münster, Germany).

### Phage host range.

The spot assay method on the surface of double-layer agar plates was used to determine the host range of the isolated phages with 63 isolates encompassing different phytopathogenic bacterial species. Challenged bacteria in the exponential growth phase (100 μL) were mixed with 3.5 mL of soft LB agar (0.7%) and poured on top of a bottom layer containing 1.5% agar. A drop of 1 μL of each amplified phage at 10^9^ PFU/mL was spotted onto bacterial lawns, followed by overnight incubation at the optimal growth temperature for each bacterial isolate. Plates were visually examined for lysis-cleared zones after 24 to 48 h, and the presence of a clear zone in the spot area was considered evidence of bacterial susceptibility to the tested phages. Three replicates were performed to ensure reproducibility of the results.

### Bacterial growth curves.

Growth curves of the strain IVIA 1317-1a in the presence and absence of each phage in liquid cultures were performed to determine the ability of the phages to lyse host bacteria. The strain IVIA 1317-1a was grown in LB broth overnight, and the OD_600_ was adjusted to 0.2 (approximately equivalent to 10^8^ CFU/mL) using fresh LB broth. A volume of 20 μL of the refreshed culture was inoculated into 178 μL of LB broth with 2 μL of each phage at 10^9^ PFU/mL (resulting in a final bacterial inoculum of 10^7^ CFU/mL). Control bacterial cultures in the absence of phage were generated in parallel. Growth curves were recorded in 96-well plates with Bioscreen C Pro (Bioscreen, Finland) equipment at 25°C, and OD_600_ measurements were acquired every 30 min across 112 h. All experiments were done in triplicate.

### DNA extraction and genome sequencing of the phages.

DNA was extracted from 10 μL of high-titer lysates by treating with DNase I to remove nonencapsidated DNA, followed by purification using the commercial DNA Clean & Concentrator 5 kit (Zymo), and DNA was quantified using Qubit. DNA was tagged for library preparation using a Nextera XT DNA library preparation kit, and samples were sequenced using the MiSeq Illumina platform (250 paired-end reads). Quality assessment of the reads was performed using the fastp program (min_length: 50; trim_qual_right: 30; trim_qual_type: mean; trim_qual_window: 10) (43). The resulting reads were assembled with SPAdes v3.13.1 (-only-assembly mode) (19). Contigs with less than 1,000 bp or corresponding to host contamination were discarded, leading to one large contig with high *k*-mer coverage per sample. Read coverage of the final contig was obtained by mapping reads using bbmap.sh (44). BLAST (20) against the nucleotide database was used to find the closest relative of each genome.

### Genomic characterization, comparative genomics, and lifestyle prediction of the phages.

Because of the transposon-based library preparation, the packaging strategy of the phages could not be predicted. Alternatively, and due to the absence of a close relative common to all the phages, the large terminase subunit, a conserved gene through all the phages, was searched by an initial annotation and fixed as the start point to reorder the genomes. The reorganized genomes were checked for assembly errors with Pilon (45) and reannotated with MultiPhATE2 (46) using Phanotate (21), Prodigal (22), and Glimmer (23) to generate a consensus gene call. BLAST (20) against the RefSeq protein database (minimum identity of 60%) was done to perform the functional analysis. Predicted ORFs without functional annotation were analyzed with CD-Search (47) using the CDD (E value threshold of 0.01) to predict conserved protein domains. The resulting annotation was manually refined, and annotated genomes were compared by BLAST (20) and represented using gggenomes (48).

To better understand the relatedness of our isolated phages between each other and previously described viruses, phages phylogenetically related to the closest relatives of isolated phages by NCBI Taxonomy were selected. To perform a single-gene phylogenetic analysis, amino acid sequences of the DNA polymerases were aligned using ClustalW (47). The best substitution model for the alignment was predicted using ProtTest 3 (49) and used to construct a maximum likelihood phylogeny with 1000 fast bootstrap pseudoreplicates in IQ-TREE (50). The best tree was represented with ITOL (51), fixing the root in the midpoint and deleting branches with a value of bootstrap lower than 90. Additionally, complete genomes of selected phages were downloaded and analyzed along with the isolated phage genomes by running VIRIDIC (52) (95% and 70% thresholds for virus delimitation in species and genera). Clusters generated by VIRIDIC were plotted using RAWGraphs (53).

Due to the similarity and phylogenetic relatedness of some of our isolated phages to temperate phages, lifestyle prediction was performed by using PhageAI (32) and Bacphlip (33).

### Data availability.

The sequences of the isolated phages vB_Xar_IVIA-DoCa1 to vB_Xar_IVIA-DoCa1-11 are available in GenBank under the accession numbers ON911538, ON911539, ON911540, ON932078, ON932079, ON932080, ON932081, ON932082, ON932083, ON932084, and ON932085, respectively.
