# Supplementary material for: Comparative Analysis of Novel Lytic Phages for Biological Control of Phytopathogenic Xanthomonas spp
Source: Microbiol Spectr. 2022 Nov 3;10(6):e02960-22. doi: 10.1128/spectrum.02960-22 (PMC9769650; doi:10.1128/spectrum.02960-22)
Supplement: Supplemental file 1 — Supplemental material. Download spectrum.02960-22-s0001.pdf, PDF file, 0.5 MB [file spectrum.02960-22-s0001.pdf]

SUPPLEMENTAL MATERIAL FOR PUBLICATION

FIG S1 Intergenomic similarities heatmap of the isolated and related phages generated by VIRIDIC.

Table S1 Bacterial strains used in this study. NA: not available.

Table S2 Functional annotation of ORFs predicted in vB_Xar_IVIA-DoCa1-11 genomes.

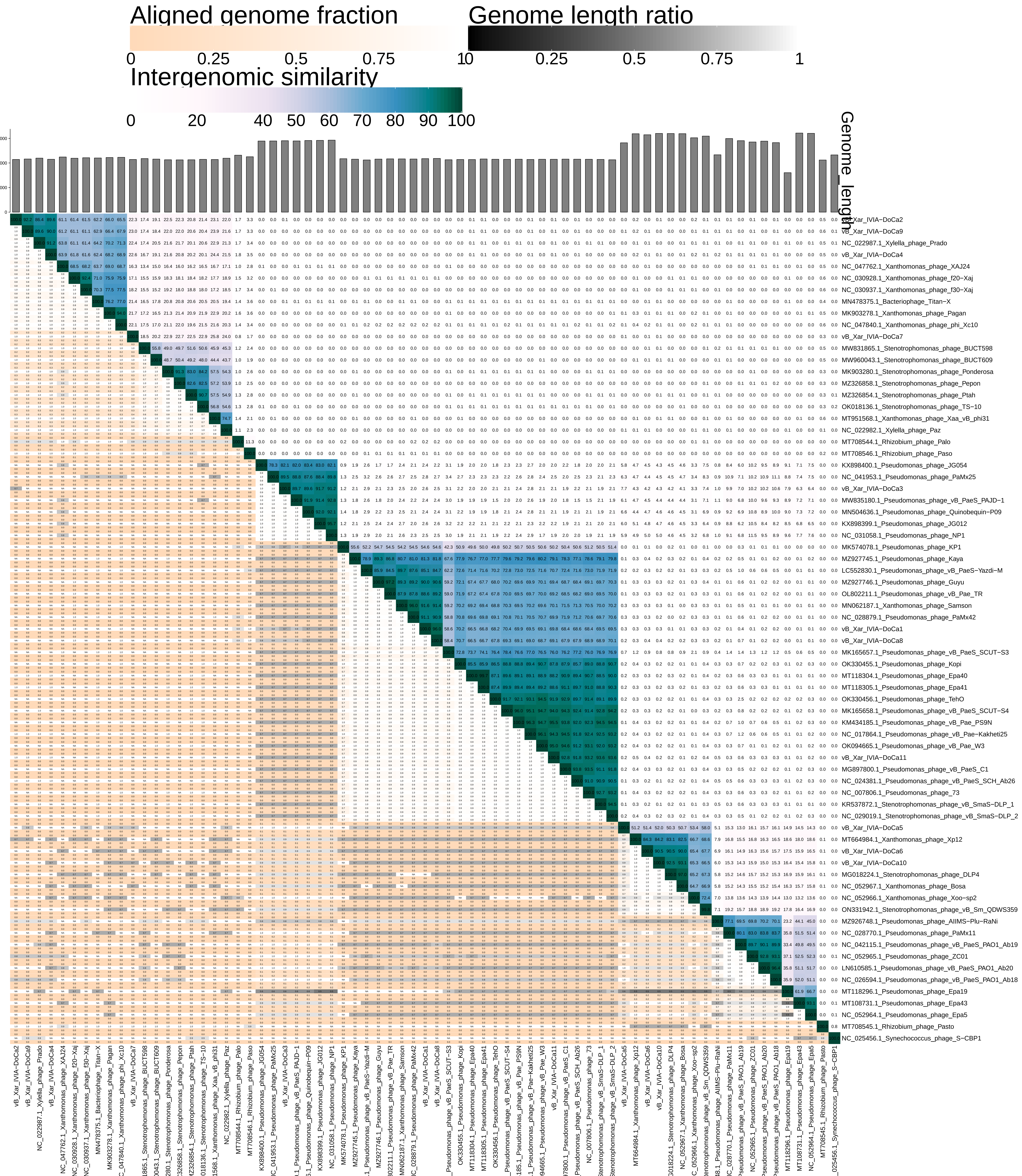
Aligned genome fraction
0
0.25
0.5
0.75
1
Genome length ratio
0
0.25
0.5
0.75
1
Intergenomic similarity
0
20
40
50
60
70
80
90
100
Genome_length
60000
40000
20000
0
vB_Xar_IVIA−DoCa2
vB_Xar_IVIA−DoCa9
NC_022987.1_Xylella_phage_Prado
vB_Xar_IVIA−DoCa4
NC_047762.1_Xanthomonas_phage_XAJ24
NC_030928.1_Xanthomonas_phage_f20−Xaj
NC_030937.1_Xanthomonas_phage_f30−Xaj
MN478375.1_Bacteriophage_Titan−X
MK903278.1_Xanthomonas_phage_Pagan
NC_047840.1_Xanthomonas_phage_phi_Xc10
vB_Xar_IVIA−DoCa7
MW831865.1_Stenotrophomonas_phage_BUCT598
MW960043.1_Stenotrophomonas_phage_BUCT609
MK903280.1_Stenotrophomonas_phage_Ponderosa
MZ326858.1_Stenotrophomonas_phage_Pepon
MZ326854.1_Stenotrophomonas_phage_Ptah
OK018136.1_Stenotrophomonas_phage_TS−10
MT951568.1_Xanthomonas_phage_Xaa_vB_phi31
NC_022982.1_Xylella_phage_Paz
MT708544.1_Rhizobium_phage_Palo
MT708546.1_Rhizobium_phage_Paso
KX898400.1_Pseudomonas_phage_JG054
NC_041953.1_Pseudomonas_phage_PaMx25
vB_Xar_IVIA−DoCa3
MW835180.1_Pseudomonas_phage_vB_PaeS_PAJD−1
MN504636.1_Pseudomonas_phage_Quinobequin−P09
KX898399.1_Pseudomonas_phage_JG012
NC_031058.1_Pseudomonas_phage_NP1
MK574078.1_Pseudomonas_phage_KP1
MZ927745.1_Pseudomonas_phage_Kaya
LC552830.1_Pseudomonas_phage_vB_PaeS−Yazdi−M
MZ927746.1_Pseudomonas_phage_Guyu
OL802211.1_Pseudomonas_phage_vB_Pae_TR
MN062187.1_Xanthomonas_phage_Samson
NC_028879.1_Pseudomonas_phage_PaMx42
vB_Xar_IVIA−DoCa1
vB_Xar_IVIA−DoCa8
MK165657.1_Pseudomonas_phage_vB_PaeS_SCUT−S3
OK330455.1_Pseudomonas_phage_Kopi
MT118304.1_Pseudomonas_phage_Epa40
MT118305.1_Pseudomonas_phage_Epa41
OK330456.1_Pseudomonas_phage_TehO
MK165658.1_Pseudomonas_phage_vB_PaeS_SCUT−S4
KM434185.1_Pseudomonas_phage_vB_Pae_PS9N
NC_017864.1_Pseudomonas_phage_vB_Pae−Kakheti25
OK094665.1_Pseudomonas_phage_vB_Pae_W3
vB_Xar_IVIA−DoCa11
MG897800.1_Pseudomonas_phage_vB_PaeS_C1
NC_024381.1_Pseudomonas_phage_vB_PaeS_SCH_Ab26
NC_007806.1_Pseudomonas_phage_73
KR537872.1_Stenotrophomonas_phage_vB_SmaS−DLP_1
NC_029019.1_Stenotrophomonas_phage_vB_SmaS−DLP_2
vB_Xar_IVIA−DoCa5
MT664984.1_Xanthomonas_phage_Xp12
vB_Xar_IVIA−DoCa6
vB_Xar_IVIA−DoCa10
MG018224.1_Stenotrophomonas_phage_DLP4
NC_052967.1_Xanthomonas_phage_Bosa
NC_052966.1_Xanthomonas_phage_Xoo−sp2
ON331942.1_Stenotrophomonas_phage_vB_Sm_QDWS359
MZ926748.1_Pseudomonas_phage_AIIMS−Plu−RaNi
NC_028770.1_Pseudomonas_phage_PaMx11
NC_042115.1_Pseudomonas_phage_vB_PaeS_PAO1_Ab19
NC_052965.1_Pseudomonas_phage_ZC01
LN610585.1_Pseudomonas_phage_vB_PaeS_PAO1_Ab20
NC_026594.1_Pseudomonas_phage_vB_PaeS_PAO1_Ab18
MT118296.1_Pseudomonas_phage_Epa19
MT108731.1_Pseudomonas_phage_Epa43
NC_052964.1_Pseudomonas_phage_Epa5
MT708545.1_Rhizobium_phage_Pasto
NC_025456.1_Synechococcus_phage_S−CBP1
